# A sustainable synthesis of polyhydroxyalkanoate from stubble waste as a carbon source using *Pseudomonas putida* MTCC 2475

**DOI:** 10.3389/fbioe.2024.1343579

**Published:** 2024-04-11

**Authors:** Neha Kukreti, Pravir Kumar, Rashmi Kataria

**Affiliations:** Department of Biotechnology, Delhi Technological University (DTU), Delhi, India

**Keywords:** alkali pretreatment, corn stover, enzymatic saccharification, PHA production, response surface methodology

## Abstract

Polyhydroxyalkanoates (PHAs) are biodegradable polymers that can be produced from lignocellulosic biomass by microorganisms. Cheap and readily available raw material, such as corn stover waste, has the potential to lessen the cost of PHA synthesis. In this research study, corn stover is pretreated with NaOH under conditions optimized for high cellulose and low lignin with central composite design (CCD) followed by characterization using Fourier-transform infrared spectroscopy (FTIR), thermal gravimetric analysis (TGA), and scanning electron microscopy (SEM). Design expert software performed further optimization of alkali pretreated corn stover for high total reducing sugar (TRS) enhancement using CCD using response surface methodology (RSM). The optimized condition by RSM produced a TRS yield of 707.19 mg/g. Fermentation using corn stover hydrolysate by *Pseudomonas putida* MTCC 2475 gave mcl-PHA detected through g*as* *c* *hromatography* *–* *t* *andem* *m* *ass* *s* *pectrometry* (GC-MS/MS) and characterization of the PHA film by differential scanning calorimetry (DSC), FTIR, and nuclear magnetic resonance (NMR). Thus, this research paper focuses on using agriculture (stubble) waste as an alternative feedstock for PHA production.

## 1 Introduction

Polyhydroxyalkanoates (PHAs) are biodegradable and renewable biopolymers that hold great potential as a sustainable alternative to petroleum-based plastics (Tanikkul et al., 2020). With plastic production reaching a staggering 448 million tons in 2015 and estimated to double by 2050, it is imperative to find ecological solutions. Synthetic plastics pose a threat to the environment and all living organisms on Earth. The appeal of petroleum-based plastics lies in their lightweight nature and cheap manufacturing, making them abundantly usable (Phanisankar et al., 2020). To address the pressing issues surrounding the generation of petroleum-based plastics and the widespread use of single-use plastic, the production of biodegradable biopolymers has become crucial (Ashby et al., 2022).

Agricultural waste, such as corn stover, is stubble waste that is left in the fields after harvesting. Stubble burning is putting fire to the straw and leftovers to remove them from the field and make the field ready for the next crop. The burning causes severe air pollution and deteriorates the air quality. This waste has the potential to be used for the production of bioplastics. Recently, there has been a growing interest in using lignocellulosic biomass as a carbon source to produce PHA (Abdurrahman et al., 2020).

Lignocellulosic biomass is highly valuable due to its renewability, abundance, and easy availability. If left unutilized, lignocellulosic biomass is often discarded in fields or burnt, leading to pollution that poses risks to human health and the environment (Saldarriaga-Hernández et al., 2020). Corn stover, a type of lignocellulosic biomass, can be effectively utilized for PHA production. It offers advantages such as economic feasibility, easy availability, no transportation issues, carbon neutrality, and sustainability. The annual global production of corn stover is nearly one billion tons. India, the third largest producer of corn globally, cultivates approximately 22.3 million metric tons of corn stover each year, making it a suitable and substantial resource for PHA production. Corn stover primarily consists of cellulose, hemicellulose, and lignin, with lignin being composed of polyphenolic compounds. The presence of lignin impedes enzymatic hydrolysis, necessitating pretreatment for efficient sugar extraction from corn stover biomass (Baadhe et al., 2014). The conventional pretreatment method involves chemical treatment with alkali or acidic conditions to remove hemicellulose and lignin, which are the major components of the resistant structure of lignocellulosic biomass. Alkali pretreatment removes lignin while maintaining the carbohydrate (cellulose and hemicellulose) portion (Cheng et al., 2020). This approach leads to higher yields of reducing sugar during enzymatic saccharification and reduces the formation of inhibitors. A combination of pretreatment methods is employed to liberate reducing sugars from corn stover, resulting in the extraction of sugars such as glucose, xylose, arabinose, galactose, and mannose (Sawant et al., 2015).

Polymers play a critical role in the global industrial economy, and the production of biopolymers from biological sources offers an alternative to reduce reliance on fossil fuels and petroleum. Polyhydroxyalkanoates (PHA) are polyesters created using carbon and nitrogen stress conditions. The production of PHA depends on the genetic makeup of the microbial strain and the substrate fed to the microorganisms depending on which the PHA produced has properties such as rigid thermoplastic in short-chain (scl-) PHA and flexible elastomers in medium-chain (mcl-) PHA. Mcl-PHA has an advantage over scl-PHA. These biopolymers can degrade into carbon dioxide and water. The fermentation costs associated with PHA production are high, and, therefore, efforts have been made to use inexpensive and renewable corn stover as a feedstock (Ashby et al., 2022). *P. putida* is the producer of mcl-PHA, which is the precursor of bioplastic (Kanavaki et al., 2021).

The present research work focuses on the utilization of corn stover, an agricultural waste product, as a carbon source for PHA production. The process involves two main steps—alkali pretreatment and enzymatic saccharification—to convert the corn stover into sugar hydrolysate. The alkali pretreatment is optimized to achieve maximum lignin removal and to enhance the depolymerization and disintegration of the tough corn stover structure. The alkali pretreated corn stover is characterized using Fourier-transform infrared (FTIR) spectroscopy, thermal gravimetric analysis (TGA), and scanning electron microscopy (SEM). The resulting residue is then subjected to enzymatic saccharification using commercial cellulase to produce high-reducing sugars. Submerged fermentation with *P*. *putida* uses the sugar hydrolysate obtained from corn stover to produce PHA. The monomers of PHA were detected using g*as* *c* *hromatography* *–* *t* *andem* *m* *ass* *s* *pectrometry* (GC-MS/MS), and the extracted PHA film was characterized using differential scanning calorimetry (DSC), FTIR spectroscopy, and nuclear magnetic resonance (NMR). Lastly, a mass balance study was conducted on a bench scale to estimate the potential large-scale production of PHA. The novelty of the work lies in using the wild type of *P. putida* MTCC 2475 strain for mcl-PHA production with a simple carbon source of corn stover for the first time.

## 2 Materials and methods

### 2.1 Collection and bioprocessing of corn stover

The corn stover used in the research study was collected from the fields of Uttar Pradesh, India. The sample was kept in a microprocessor-controlled oven (Metrex Scientific Instruments, New Delhi, India) at 35°C for drying until it reached a constant weight, then cut into 2–4 cm size pieces, pulverized through a grinder (Bajaj GX3701), sieved to 1-mm mesh, and kept in airtight containers for further experimental analysis. All chemicals used in the study were obtained from Thermo Fisher Scientific and Sisco Research Laboratories (SRL).

### 2.2 Compositional analysis of corn stover

Structural carbohydrates and lignin were determined using the National Renewable Energy Laboratory (NREL) protocol (Sluiter et al., 2012). The crystalline cellulose content was estimated using the Updegraff reagent method (Bauer and Ibá, 2014). The ash content was determined using some modifications to the method described by Lizotte et al. (2015), and a 1 g sample was weighed in the crucible and kept in a muffle furnace at 550°C for 8 h.

### 2.3 Optimization of alkali pretreatment conditions of corn stover by response surface methodology using Minitab

Central composite design (CCD) was used to optimize two parameters: cellulose and lignin content. The design consisted of 13 sets of experiments involving five replicates at center points. Two parameters were varied: A) NaOH concentration with −1 (0.25%), 0 (1.625%) and +1 (3%); B) Time in minutes with −1 (15), 0 (45), and +1 (75) minutes. The alkali pretreatment optimization for high cellulose and low lignin was performed in an autoclave at 121°C for all 13 run orders.

A 10 g sample was placed in 500-mL Borosil reagent bottles, each containing different alkali concentrations: 0.25%, 1.625%, and 3% NaOH (w/v) and time: 15 min, 45 min, and 75 min with corresponding pH values of 10.8, 12.6, and 13.7. After pretreatment, the solid and liquid parts of the biomass were separated using muslin. The solid biomass was neutralized with Milli-Q water, and the recovered liquid was neutralized with 1N HCl. The liquid hydrolysate was then filtered using a 0.2-micron syringe filter, and the solid biomass was dried in an oven at 40°C until a constant weight was achieved and then stored for further experiments. The recovered solid biomass was used to analyze cellulose and total lignin content (Baadhe et al., 2014). The cellulose content in the solid biomass was determined using the Updegraff method, and total lignin was analyzed following the NREL protocol described by Sluiter et al. (2012).

### 2.4 Characterization of corn stover before and after pretreatment

FTIR was conducted using the FTIR spectrophotometer (Perkin Elmer Frontier) for functional groups in the frequency range of 4,000–400 cm^−1^. The pellet was prepared using KBr (FTIR grade), and the sample was thoroughly crushed and mixed in a mortar and further pressed using a pellet press machine (Fan et al., 2012). Thermal gravimetric analysis (TGA) was conducted to determine the thermal stability of untreated and treated samples used for experiments using a thermogravimetric analyzer (Perkin Elmer/TGA 4000) with a maximum temperature of 900°C and heating rate of 10°C min−1 under a nitrogen atmosphere. The weight change of corn stover biomass was recorded following temperature and time. A 2 g sample of dried powder was placed in the crucible in the analyzer (Velázquez-Martí et al., 2018). The morphology of untreated and treated samples was determined using a scanning electron microscope (ZEISS EVO 18 Research) (Shamala et al., 2009).

### 2.5 Enzymatic saccharification of alkali-pretreated corn stover

The alkali pretreated biomass with high cellulose and low lignin content was subjected to enzymatic saccharification using commercial cellulase (Meicellase, from *Aspergillus niger,* 13000CMC U/g) from Sisco Research Laboratories. One study used 20 U/g of commercial cellulase for enzymatic saccharification (Wu J. et al., 2021). The experiments were designed for optimization of conditions with biomass loading % and enzyme U. Total reducing sugar (TRS) was estimated by the di-nitro salicylic acid (DNS) method (Gusakov et al., 2011), and further corn stover hydrolysate was stored at 4°C for its use in PHA production.

#### 2.5.1 Optimization of alkali pretreated biomass for enhanced enzymatic saccharification with commercial cellulase for maximum TRS yield

A study was conducted using Design Expert to optimize enzymatic saccharification conditions for high TRS from alkali-pretreated corn stover. A central composite design with two factors, biomass loading (%) and enzyme units (U), was employed to analyze enzymatic saccharification for total reducing sugar (TRS). The selected variables were represented by alpha values, indicating the highest, central, and lowest points (−alpha, −1, 0, +1, and +alpha). Biomass loading ranged from 1.37% to 5.62%, and enzyme units varied from 11.7 U to 68.28 U. The temperature was set at 50°C, with a 0.05 M citrate buffer at pH 4.8, agitation at 200 rpm, and a duration of 72 h as fixed parameters. All experiments were conducted in triplicate to assess the consistency of the results. The resulting sugars were used in the calculation of saccharification by the following equation:

$$Saccharification=\frac{Total\;reducing\;sugars\;\left(g/L\right)\times 0.9\times dilution\;factor}{biomass\;\left(g\right)}.$$

The factor 0.9 is taken as it converts polysaccharides to monosaccharides, considering the water uptake in the hydrolysis process (Alrumman, 2016).

### 2.6 Microbial strain, media preparation, and bacterial growth curve

*Pseudomonas putida* MTCC 2475 was obtained from the Microbial Type Culture Collection and Gene Bank (MTCC), Chandigarh, India. Luria-Bertani (LB) agar containing 10 g/L tryptone, 5 g/L yeast extract, 10 g/L NaCl, and 15 g/L agar was used for cell maintenance. The seed culture composition included 10 g/L tryptone, 5 g/L yeast extract, and 10 g/L NaCl. The pH of the seed culture was adjusted to 7 and autoclaved at 121°C for 15 min. The microbial strain (*P. putida* MTCC 2475) was inoculated into the seed culture, and the mixture was placed in an orbital shaker at 30°C and 150 rpm for 16 h until the optical density (O.D.) at 600 nm reached 0.8.

Four different types of media conditions were tested: 1) LB broth with tryptone, 10 g/L; sodium chloride, 5 g/L; yeast extract, 5 g/L; 2) Only hydrolysate, 10 g/L; 3) Modified media with synthetic glucose, 10 g/L; sodium chloride, 5 g/L; ammonium sulfate, 1 g/L; and 4) Modified media with hydrolysate, 10 g/L; sodium chloride, 5 g/L; ammonium sulfate, 1 g/L. LB broth ***(A)***, hydrolysate only ***(B)***, modified media with synthetic glucose ***(C)***, and modified media with glucose replaced by hydrolysate ***(D)*** were inoculated with a 1% bacterial suspension to compare bacterial growth. The bacterial suspensions were incubated at 37°C, and the growth curves, dry cell weights, and sugar consumptions were measured at 2-h intervals. Sugar consumption was analyzed using the di-nitro salicylic method (Gusakov et al., 2011).

### 2.7 PHA production using corn stover hydrolysate

The production media experiments were conducted in 2-L shaking flasks with nitrogen stress containing 1 L of media, comprising 1 g/L ammonium sulfate, 5 g/L sodium chloride, and 10 g/L corn stover hydrolysate. Corn stover hydrolysate, obtained after enzymatic saccharification, was the carbon source. Ammonium sulfate [(NH4)_2_SO_4_] was used as the nitrogen source, while sodium chloride (NaCl) was added to maintain osmotic balance. Sodium chloride and ammonium sulfate were sterilized in an autoclave at 121°C for 20 min, and the hydrolysate was sterilized through a 0.2-µ syringe filter before being added to the flask prior to inoculation. To maintain a 10: 1 C/N ratio, 1 g/L of ammonium sulfate was added to the flask.

The flasks were inoculated with 1% of an overnight seed culture of *P. putida* and incubated for 24 h, 36 h, 48 h, 60 h, and 72 h at 30°C and 150 rpm. Bacterial cells were harvested by centrifugation, and the resulting pellets were washed with Milli-Q water and dried until a constant weight was achieved.

### 2.8 Extraction of PHA and GC-MS/MS for monomer composition

The methanolysis method was used to extract PHA using 20 mg of dried cells. The cells were mixed with 2 mL of chloroform for extraction in a 15 mL Borosil-sealed culture tube. To this mixture, 2 mL of acidified methanol (85% v/v methanol and 15% v/v sulfuric acid) was added, and the tube was incubated at 100°C for 140 min. During this process, the polymer PHA fragmented into fatty acids methyl esters, which are the monomeric components. After cooling, 2 mL of Milli-Q water was added for organic phase separation, as described by Mahato (2021). The resulting organic layer was transferred to another vial, filtered through a 0.2-µ syringe filter, and stored in GC vials.

Subsequently, the organic layer was analyzed using GC-MS/MS (Agilent GC7000D/TQ) to identify the monomers of PHA. A 2 µL injection volume was used in the gas chromatograph, with inert helium serving as the carrier gas at a flow rate of 2 mL/min. The oven temperature was set at 200°C, and N-hexane was used as a blank. PHA monomer compounds were identified using the NIST 17 library database (Mahato, 2021).

### 2.9 Characterization of the extracted PHA film

Thermal analysis of the extracted PHA film was conducted using DSC. A 5 mg sample was placed in a crucible and loaded into the sampler port of a PerkinElmer DSC 8000 instrument. The PHA film was heated from 30°C to 250°C at a rate of 10°C/min, with nitrogen gas used for purging, following the method described by Dartiailh et al. (2021).

For structural analysis, FTIR was employed to identify functional groups in PHA. A 1 mg extracted sample was mixed with KBr to form a pellet. The PHA polymer was then analyzed using a PerkinElmer Frontier FTIR spectrophotometer in the range of 4,000–450 cm^−1^, as detailed by Mahato (2021).

Additionally, ^1^H NMR spectroscopy was performed using a Bruker 500 MHz spectrometer to analyze both the chemical structure and monomer composition of the extracted PHA film. For this analysis, 4 mg of the extracted PHA film was dried and dissolved in 600 µL of CDCl_3_ (Dartiailh et al., 2021).

## 3 Results and discussion

### 3.1 Composition of corn stover

Corn stover was characterized for composition % (w/w) as follows: cellulose, 41.67%; total lignin, 24.59%; ash, 11.26%; and other components, 3.51%, as indicated in Table 1. In other research studies, untreated corn stover exhibited a cellulose content of 36.5%, a hemicellulose content of 22.1%, and a lignin content of 18.8%, highlighting its high carbohydrate content available for bioproduct generation. Thus, corn stover is a key lignocellulosic biomass for sugar recovery and value-added product generation (Yang et al., 2016; Li et al., 2020).

**TABLE 1:** Composition of corn stover.

Parameter	Corn stover % (w/w)
1. Total structural carbohydrate	59.60 ± 0.13
2. Cellulose	41.67 ± 0.59
3. Total lignin	24.59 ± 0.34
4. Acid-insoluble lignin	22.05 ± 0.34
5. Acid-soluble lignin	02.54 ± 0.00
6. Ash	11.26 ± 0.12

Results are expressed as mean standard, followed by standard deviation.

### 3.2 Optimization of alkali pretreated corn stover by response surface methodology

Response surface methodology is a mathematical and analytical tool to analyze the data using models. It has optimization approaches for setting variables and obtaining responses with maximum and minimum values. Central composite design (CCD) is a factorial response surface design with center and axial points to analyze the curvature (Sharif et al., 2023).

Alkali pretreatment with sodium hydroxide can increase the effectiveness of enzymatic saccharification of corn stover for PHA production. Therefore, CCD was used to optimize cellulose and lignin content in corn stover by optimizing alkali concentration (%) and time (minutes) in the pretreatment process. The responses were cellulose % and lignin content % present after the alkali pretreatment, as shown in Table 2. Comparing the cellulose and lignin content before and after the pretreatment, the cellulose content in untreated corn stover was found to be 41.69%, and the cellulose content in corn stover was 83.58% after pretreatment with 3% NaOH (w/v) for 15 min. Thus, the alkali pretreatment resulted in an increased cellulose content. Furthermore, the lignin content in untreated corn stover was 24.59%, and after pretreatment with 3% NaOH (w/v) for 15 min, the lignin content was 7.17%. There is a decrease in lignin content as the long-term NaOH chemical pretreatment, along with high temperatures, causes exposure of free hydrogen bonds in cellulosic structure, which causes an increase in affinity with water and accumulation of corn stover particle agglomerates. Sodium hydroxide pretreatment also solubilizes and extracts lignin from lignocellulosic biomass by affecting the acetyl and ester groups, decreasing the degree of polymerization, and breaking the bonds between lignin and other carbohydrate polymers. Therefore, alkali pretreatment is an important step to increase cellulose digestibility and improve lignin solubilization (Venturin et al., 2018; Klongklaew et al., 2023).

**TABLE 2:** Central composite design and the response for cellulose (%) and lignin (%) with different alkali concentrations (%) and time (min).

Run order	Alkali concentration (%)	Time (minutes)	Solid recovery (g)	Cellulose (%)	Lignin (%)
1	3 (+1)	15 (−1)	55.11 ± 0.02	83.58 ± 0.15	7.17 ± 0.49
2	3 (+1)	45 (0)	50.67 ± 0.26	71.23 ± 0.65	6.54 ± 0.30
3	1.625 (0)	45 (0)	62.22 ± 0.14	81.02 ± 1.88	11.92 ± 0.07
4	1.625 (0)	45 (0)	62.41 ± 0.02	71.59 ± 0.94	11.98 ± 0.15
5	3 (+1)	75 (+1)	48.8 ± 0.85	56.23 ± 0.35	4.69 ± 0.27
6	1.625 (0)	75 (+1)	58.8 ± 0.48	68.80 ± 0.36	11.32 ± 0.51
7	0.25 (−1)	15 (−1)	77.28 ± 0.69	47.03 ± 0.63	22.4 ± 0.26
8	1.625 (0)	15 (−1)	65.81 ± 0.77	79.11 ± 1.81	13.17 ± 0.24
9	0.25 (−1)	75 (+1)	68.60 ± 0.92	53.65 ± 0.40	21.85 ± 0.10
10	1.625 (0)	45 (0)	63.58 ± 0.02	72.89 ± 1.06	11.39 ± 0.02
11	1.625 (0)	45 (0)	62.60 ± 0.73	75.90 ± 1.98	11.64 ± 0.50
12	0.25 (−1)	45 (0)	72.03 ± 0.38	46.51 ± 0.33	22.03 ± 0.15
13	1.625 (0)	45 (0)	63.48 ± 0.78	74.67 ± 0.02	10.68 ± 0.60

Amounts of solid biomass recovered after different alkali pretreatment conditions are given in Table 2. The solid recoveries after the pretreatment decrease with increasing alkali concentration and time as the pretreatment conditions involve increasing NaOH solutions, which increase the solubility of lignin and carbohydrate components and further lead to loss of solid biomass in the pretreatment process. Alkali solutions partially dissolve the solid cellulose component and decrease the solid biomass recovery. Alkali pretreatment also shows structural modifications in the solid biomass after pretreatment, like cell wall damage or microfibril degradation, which cause difficulty in recovering the solid biomass. The SEM images of solid residue remaining after the pretreatment conditions with 1.625% and 3% NaOH (w/v) show a rough and exposed structure, whereas untreated corn stover shows a smooth surface, as shown in Figure 2C.

The optimized condition obtained for high cellulose and low lignin content after alkali pretreatment was at 3% NaOH (w/v) for 15.9 min. The optimized condition, carried out with 100 g corn stover, had a solid recovery of 55.8 g with 82.91% cellulose and 7.53% of the lignin content, as shown in Table 3. Yang et al. (2022) reported the mechanochemical pretreatment combined with alkaline pretreatment of corn stover. The highest glucose yield was 91.9% at 3% NaOH and ball milling for 10 min. The optimal condition had 44.4% lignin removal and 86.6% cellulose retention. According to Gao et al. (2020), pretreatment of silvergrass at 4% NaOH concentration resulted in cellulose removal of 45.2%, hemicellulose removal of 74.4%, and lignin removal of 92.7%. On increasing the concentration from 4% to 5% NaOH, hydrolysis efficiency decreased due to an increase in cellulose crystallinity. There is no change in lignocellulosic biomass degradation by varying the solid-to-liquid ratio (*p* > 0.05). Nor is there any change in cellulose degradation by increasing the residence time (*p* > 0.05). The lignin removal was enhanced by increasing the time to 90 min (*p* > 0.05). Regression equations obtained by RSM are given as

$$Regression\;equation\;for\;Lignin\;\left(\%\right)=25.251-9.231\;NaOH-0.0339\;Time+1.213\;NaOH\times NaOH+0.000281\;Time\times Time-0.01152\;NaOH\times Time,$$

**TABLE 3:** Experimental and predicted values of cellulose (%) and lignin (%).

NaOH (%)	Time (minutes)	Cellulose (%)		Lignin (%)	
		Experimental	Predicted	Experimental	Predicted
3	15.9	82.91	83.57	7.53	7.45

Regression equation for cellulose (%) = 33.72 + 43.04 NaOH +0.166 Time–8.005 NaOH × NaOH–0.00004 Time × Time–0.2063 NaOH × Time.

A *p*-value > 1 indicates that the model terms are not significant. In the case of the studied factors, the maximum F-value in cellulose and lignin implies the most significant variable, and the lowest F-value implies the least significant factor. The regression coefficient (R-square value) of the lignin % model is 99.52%, the predicted R-square value is 99.17%, and the adjusted R-square value is 98%. The regression coefficient (R-square value) of the cellulose % model is 96.59%. The predicted R-square value is 94.15%, and the adjusted R-square value is 89.86%. Analysis of variance (ANOVA) of the results for the response of high cellulose (%) and low lignin (%) content from alkali pretreatment of corn stover is shown in Table 4.

**TABLE 4:** **a)** Analysis of variance (ANOVA) of the results for the response of cellulose (%) content from alkali pretreatment of corn stover. (**b)** ANOVA of the results for the response of lignin (%) content from alkali pretreatment of corn stover.

(a)						
Source	SS	Df	Mean square	F-value	*p*-value	
**Model**	1871.73	5	374.346	39.64	0.00	Significant
Linear	840.67	2	420.336	44.51	0.00	
NaOH	679.47	1	679.47	71.95	0.00	
Time	161.20	1	161.202	17.07	0.004	
Square	741.38	2	370.688	39.25	0.00	
NaOH × NaOH	632.55	1	632.549	66.98	0.00	
Time × Time	0.00	1	0.004	0.00	0.984	
Two-way interaction	289.68	1	289.68	30.67	0.001	
NaOH × Time	289.68	1	289.68	30.67	0.001	
Error	66.11	7	9.444			
Lack of fit	13.12	3	4.374	0.33	0.805	
Pure error	52.98	4	13.246			
**Model statistics**						
R2	96.59					
Adj-R2	94.15					
Pred-R2	89.86					
**(b)**						
**Source**	**SS**	**Df**	**Mean square**	**F-value**	***p*-value**	
Model	406.11	5	81.222	288.85	0.000	Significant
Linear	386.579	2	193.29	687.4	0.000	
NaOH	382.561	1	382.561	1,360.51	0.000	
Time	4.018	1	4.018	14.29	0.007	
Square	18.628	2	9.314	33.12	0.000	
NaOH × NaOH	14.525	1	14.525	51.66	0.000	
Time × Time	0.177	1	0.177	0.63	0.453	
Two-way interaction	0.902	1	0.902	3.21	0.116	
NaOH × Time	0.902	1	0.902	3.21	0.116	
Error	1.968	7	0.281		0.000	
Lack of fit	0.86	3	0.287	1.03	0.467	
Pure error	1.108	4	0.277		0.000	
**Model statistics**						
R2	99.52					
Adj-R2	99.17					
Pred-R2	98					

**FIGURE 1:**
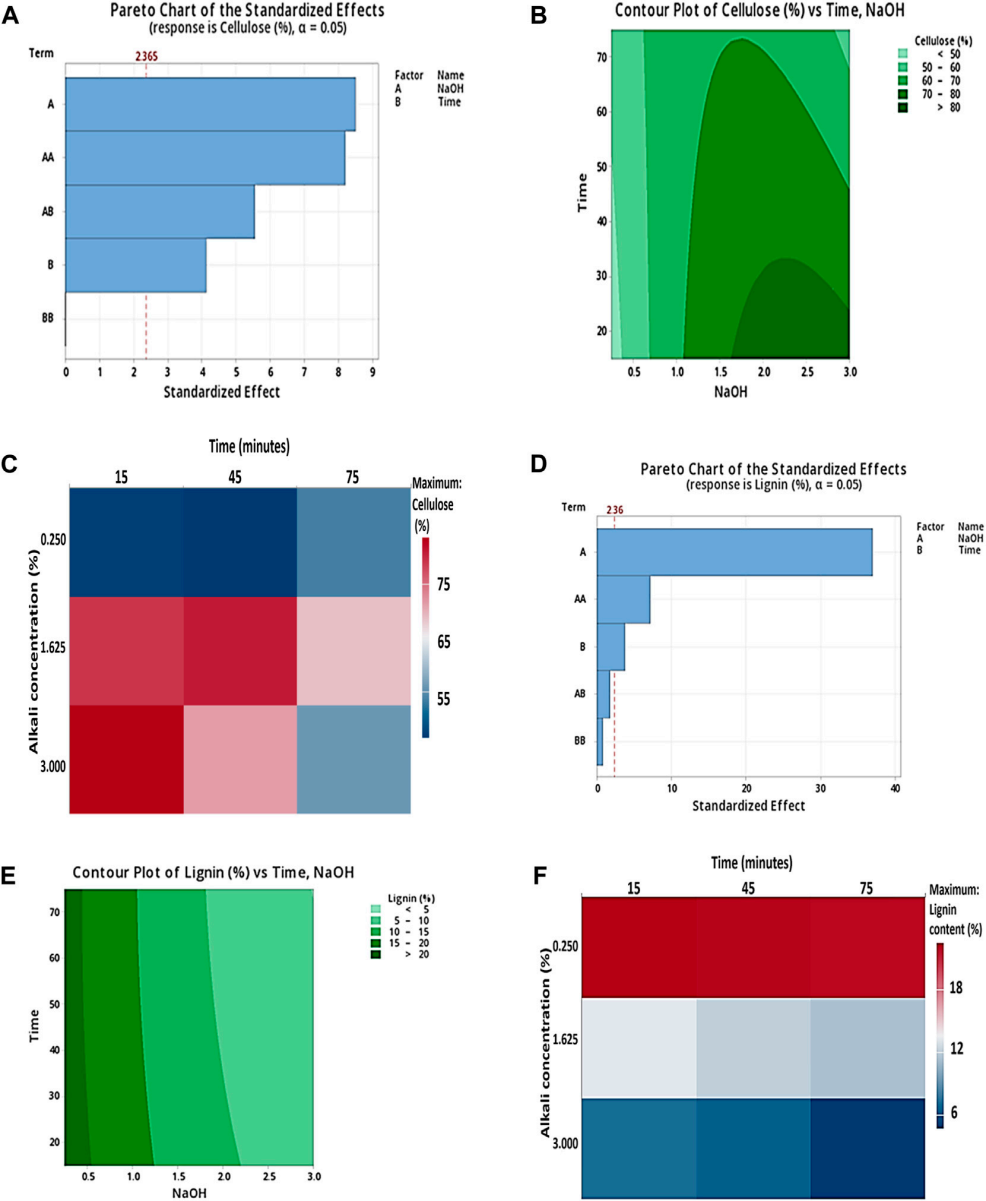
**(A)** Pareto charts of the standardized effect with two factors, NaOH and time, showing the response of cellulose (%); **(B)** response contour plots for alkaline pretreatment of corn stover describing the interaction of time (min) and 3% NaOH (w/v), that is, the effect on cellulose (%) vs. time and NaOH; and **(C)** response heat maps for alkaline pretreatment of corn stover showing the interaction between 3% NaOH (w/v) and time (min) with maximum cellulose content (%), respectively. **(D)** Pareto charts of the standardized effect with two factors, NaOH and time, showing the response of lignin (%); **(E)** response contour plots for alkaline pretreatment of corn stover describing the interaction of time (min) and 3% NaOH (w/v), that is, the effect on lignin (%) vs. time and NaOH; and **(F)** response heat maps for alkaline pretreatment of corn stover showing the interaction between 3% NaOH (w/v) and time (min) with minimum lignin content (%), respectively.

Figures 1A,D describe the Pareto charts of the standardized effects with responses (lignin and cellulose %). The Pareto chart shows a reference line to detect which factors are statistically significant, and the bars that cross the reference line are significantly important. In the Pareto chart in Figure 1A, the reference line 2.36 crosses factors A, AA, and B, and in Figure 1D, reference line 2.365 crosses factors A, AA, AB, and B, indicating these are statistically significant factors. Therefore, the factors represent statistical significance at the 0.05 level with current model terms. Figures 1B,E depict the response contour plots of cellulose % concerning NaOH and time and lignin (%) concerning NaOH and time. Figure 1B shows the high cellulose content area in dark green with nearly 1.7%–3% NaOH (w/v) and time (10–30) minutes. In Figure 1E, less lignin content is shown in light green with no effect of time. Figures 1C,F show the response heat maps with maximum cellulose and less lignin content in 3% NaOH (w/v) for 15 min. In this research study, lignin content decreased to 7.17%, lignin removal was 70.84%, and cellulose content increased to 83.58% in corn stover after the alkali pretreatment, which indicates the major role of alkaline pretreatment in removing lignin from corn stover and exposing a large portion of biomass for effective enzymatic saccharification.

### 3.3 Characterization of corn stover before and after pretreatment

Untreated and alkali-pretreated corn stover was characterized to understand the physiochemical changes before and after alkali pretreatment. Changes in cellulose crystallinity, thermal characteristics, and surface elemental composition were measured.

#### 3.3.1 Fourier-transform infrared spectroscopy

**FIGURE 2:**
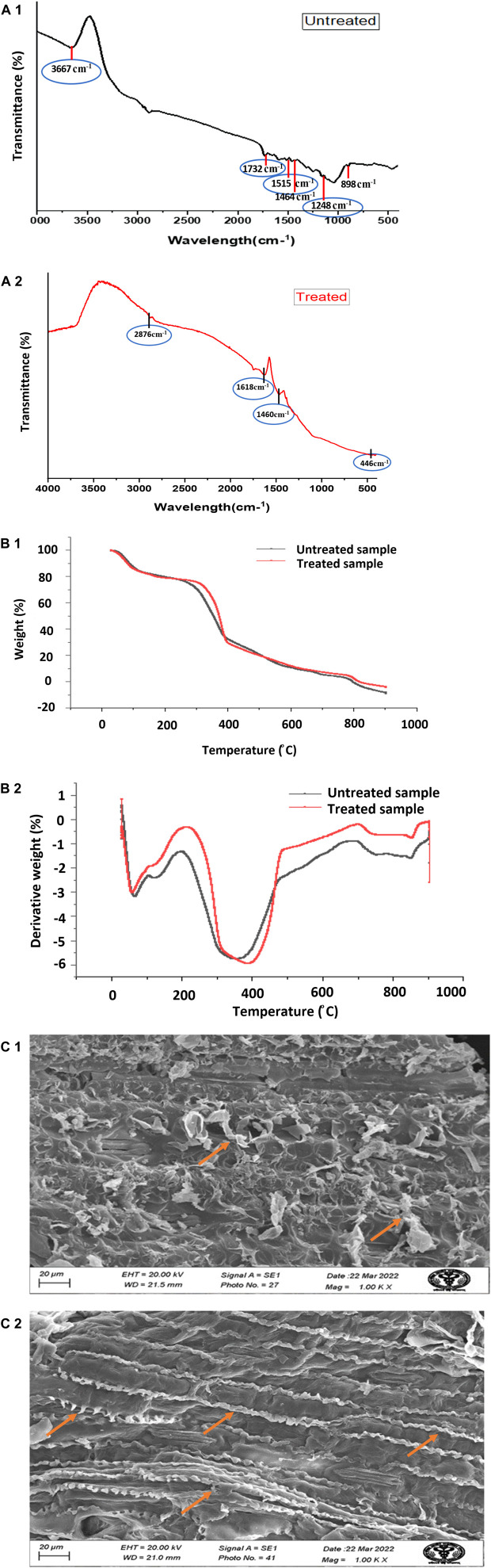
(Continued). **(A1, A2)** FTIR spectra of untreated and treated-optimized alkali pretreated corn stover; **(B1, B2)** TGA and DGA, respectively, of untreated and treated with 3% NaOH (w/v) for 15.9 min optimized corn stover; and **(C1, C2)** SEM analysis images of the untreated and pretreated optimized sample at 1,000×.

Figure 2A shows the FTIR spectra recorded between 4,000 and 400 cm^−1^. The two FTIR spectra of the untreated corn stover sample and the sample that received the optimized treatment of 3% NaOH (w/v) for 15.9 min allow study of the structural changes in corn stover, changes in crystallinity, chemical functional groups, the structure of lignin, and the bonding of carbohydrate and lignin complex. An increase in width and symmetry between 3,200 cm^−1^ and 3400 cm^−1^ indicates dissociation of the cellulosic structure, whereas changes in the peak intensity at 2915 cm^−1^ indicate–CH_2_ stretching and rupture of cellulose (Sharma et al., 2016). A decrease in intensity at approximately 1652 cm^−1^ demonstrates lignin removal, and this peak is associated with the stretching vibration of aromatic rings and phenyl ester side chain C=O bonds of lignin. The peak at 1056 cm^−1^ indicates the removal of amorphous cellulose. The peak around 1732 cm^−1^ is due to C=O stretching vibration, the peak at 1515 cm^−1^ is due to C=C aromatic symmetrical stretching, the peak at 1464 cm^−1^ is due to HCH and OCH in-plane bending vibration, the peak at 1,248 cm^−1^ is due to G ring stretching, the peak at 1161cm^−1^ is due to stretching of unconjugated C-O bonds, and the peak at 1,039 cm^−1^ is due to aromatic C-H deformation (Woźniak et al., 2021). Therefore, band intensities are reduced after alkali pretreatment. FTIR analysis of the treated corn stover sample shows the disappearance of many peaks due to the removal of lignin and hemicellulose components from the biomass after NaOH pretreatment (Fan et al., 2012).

#### 3.3.2 Thermal stability analysis

Figure 2B shows the TGA and DTG curves, which show the thermal characteristics of untreated and alkali-optimized treated corn stover. In untreated corn stover, the sample started with 99.441% initial weight at 29.62°C and the weight reduced to 50.984% at 352.21°C, and at 796.54°C, the weight was 0.024% of the initial weight. In the optimized sample at 3% NaOH (w/v) for 15 min, the initial sample weight was 99.121% at 29.58°C, and the weight was reduced to 50.927% at 366.76°C and further to 0.000% at 682.97°C. The relationship is drawn between the change in weight and temperature to check the thermal behavior. As in Ellison et al. (2023), the first stage below 100°C in both untreated and treated corn stover is due to moisture loss. The second stage, between 200°C and 400°C, represents nearly 55%–60% weight loss due to the degradation of carbohydrates (hemicellulose and cellulose), and it is the fastest region of weight loss. The decomposition beyond 400°C represents the mass loss due to lignin residues. The lignin residue, being less in the treated sample, became more susceptible to fast thermal degradation (Ellison et al., 2023).

#### 3.3.3 Scanning electron microscopy

Figure 2C shows SEM analysis of untreated and optimized treated corn stover samples. An untreated corn stover sample shows the compact structure of lignocellulosic biomass, which is covered with lignin and forms a smooth structure. After pretreatment with 3% (w/v) NaOH, lignin had broken down and condensed in the form of spiral sheets that were not present in the untreated samples. The chemical hydrolysis has created a large surface area on the biomass for better enzyme saccharification, and the structure has loosened up. SEM results explain that NaOH pretreatment was effective in removing the lignin from corn stover biomass (Mpho et al., 2020; Yang et al., 2022). The biomass characterization results demonstrate that the alkali solution has disrupted the lignin component and exposed cellulose and hemicellulose in corn stover, making the enzymes accessible during enzymatic saccharification. After the pretreatment, the enzymes can efficiently catalyze the saccharification process, producing a higher sugar yield. Therefore, alkali pretreatment is an important step in the conversion of corn stover to PHA (Sabeeh et al., 2020).

### 3.4 Enzymatic saccharification of alkali-pretreated corn stover for enhanced TRS recovery

Alkali pretreatment was performed as it helps partially break down lignin and carbohydrate components in corn stover, making it more accessible for the enzymes. Enzymatic saccharification uses enzymes for the complete conversion of cellulose into glucose. This step was also optimized for high TRSs in which 55.8 g of alkali-pretreated corn stover was digested with commercial cellulase (Meicellase) from *A. niger.* Thirteen sets of experimental designs were obtained by RSM with two parameters of biomass loading (%) and enzyme units U), and the response was total reducing sugar (%), as shown in Table 5. The maximum TRS concentration reached 719.22 mg/g. The minimum TRS concentration was 94.76 mg/g at 5% biomass loading and 20 U enzyme. The TRS was the lowest when the biomass loading was high, and the enzyme concentration was low. The effect of enzyme concentration and biomass loading were examined to analyze whether higher enzyme concentration leads to higher TRS recovery. Modenbach et al. (2017) reported lower cellulose conversion at 60 FPU/g than at 5.2 FPU/g at 5% biomass loading. Increasing the solid loading also decreased the cellulose conversion, and a higher enzyme-to-substrate ratio limits the diffusion process.

**TABLE 5:** Central composite design and responses of TRS (mg/g) with different biomass loading (%) and enzyme units U).

Run order	Biomass loading (%)	Enzyme units U)	Total reducing sugar (mg/g)
1	3.5 (0)	40 (0)	339.49
2	1.37 (−alpha)	40 (0)	719.22
3	3.5 (0)	40 (0)	360.74
4	2 (−1)	60 (+1)	540.08
5	3.5 (0)	40 (0)	343.76
6	3.5 (0)	68.28 (+alpha)	294.90
7	5 (+1)	20 (−1)	94.76
8	3.5 (0)	11.715 (−alpha)	209.70
9	5.62 (+alpha)	40 (0)	94.87
10	3.5 (0)	40 (0)	365.46
11	3.5 (0)	40 (0)	355.96
12	5 (+1)	60 (+1)	143.66
13	2 (−1)	20 (−1)	496.46

Delignified lignocellulosic biomass gives higher glucose concentration than non-delignified lignocellulosic biomass as the lignin component hampers the enzymatic hydrolysis. Increasing the enzyme concentration did not increase glucose concentration as the higher enzyme results in adsorption on the surface of the substrate, restricting the diffusion through the lignocellulosic biomass structure. Alkaline delignification enhances enzymatic saccharification. According to Klongklaew et al. (2023), the pretreated cornstover yielded increased sugar as the enzyme concentration increased from 10–40 FPU/g. The maximum sugar was released when the material was treated with 40 FPU/g after pretreatment with 1% sulfuric acid. Li et al. (2019) reported 100 g/L glucose by treating corn stover with 72 wt.% sulfuric acid, but concentrated acid pretreatment yields high sugar and generates high inhibitory compounds. Thus, alkali pretreatment is better for the delignification of lignocellulosic biomass. In addition, biomass pretreatment is important before enzymatic saccharification due to the recalcitrant and tough nature of lignocellulosic biomass. Pretreatment provides easy access so that the enzymes can act on the biomass. After enzymatic saccharification, cellulose converts to C-6 hexose sugars (e.g., glucose), and hemicellulose converts to C-5 pentose sugars (xylose and arabinose). As reported, corn stover hydrolysate is mainly composed of glucose and xylose (Wu Y. et al., 2021). Under optimized conditions, 707.19 mg/g of corn stover hydrolysate was recovered. It was stored at −20°C for its use in PHA production, as shown in Table 6.

$$Regression\;equation\;of\;coded\;levels\;TRS\left(\frac{mg}{g}\right)=714.2-\;216.3\;Biomass\;loading\;\%+11.78\;Enzyme\;U+10.65\;Biomass\;loading\;\%\times Biomass\;loading\;\%-0.1325\;Enzyme\;U\times Enzyme\;U+0.044\;Biomass\;loading\;\%\times Enzyme\;U.$$

**TABLE 6:** Experimental and predicted values of TRS (mg/g).

Biomass loading (%)	Enzyme (U)	TRS (mg/g)	
		Experimental	Predicted
1.37	44.85	707.195	702.316

**FIGURE 3:**
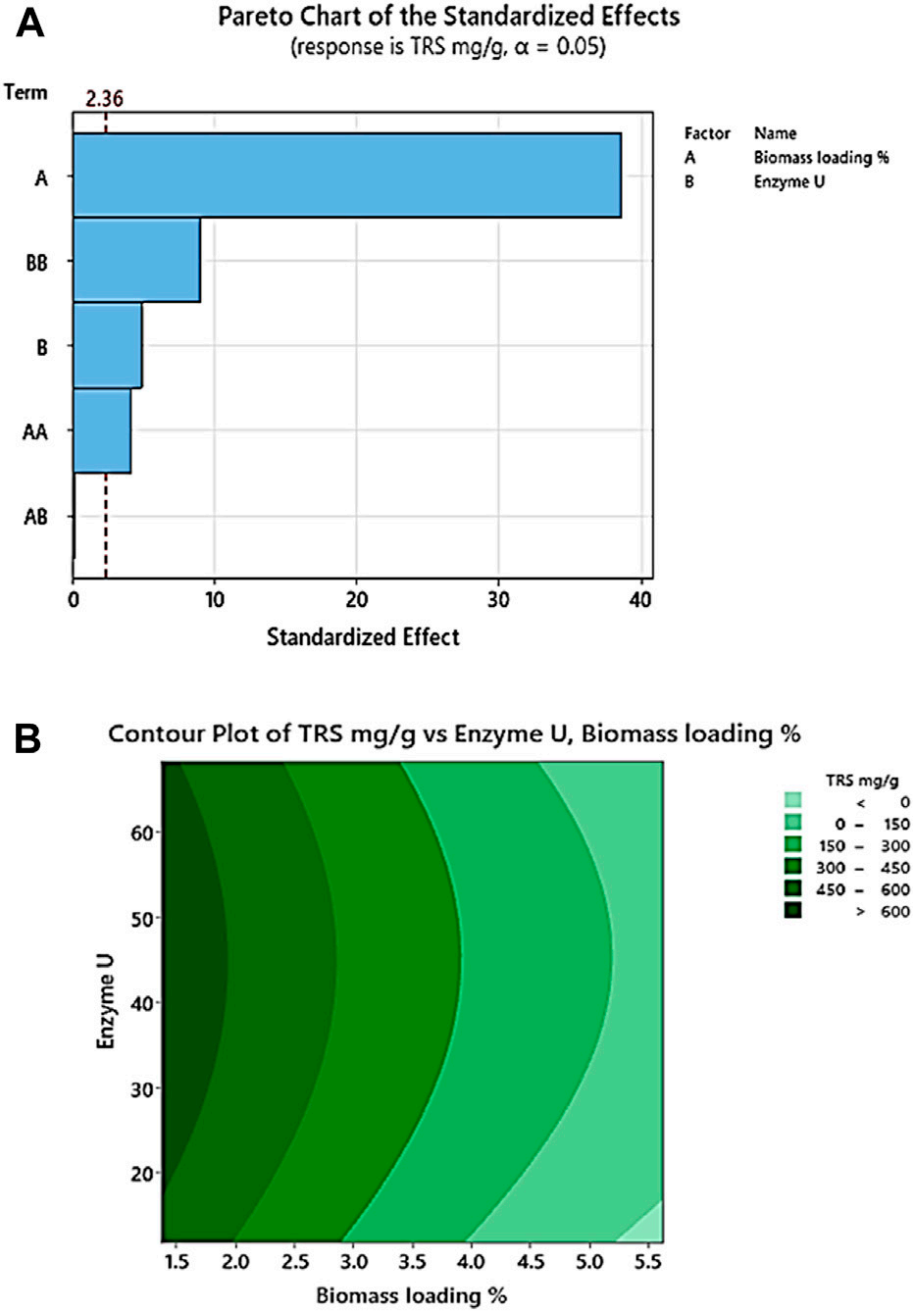
**(A)** Pareto chart of the standardized effect with two factors, biomass loading (%) and enzyme (U), showing response TRS (mg/g). **(B)** Response contour plot for enhanced TRS (mg/g) with 3% NaOH (w/v) pretreated corn stover describing the interaction of enzyme (U) and biomass loading (%).

According to the two factors studied, biomass loading % and enzyme U, biomass loading × biomass loading, and enzyme U × enzyme U are significant as their *p*-values are less than 0.100. Biomass loading % plays the most significant role as its F-value is maximum. The regression coefficient (R-square value) of the TRS (mg/g) model is 99.57%, the predicted R-square value is 97.64%, and the adjusted R-square value is 99.26%. Therefore, we can infer that pretreated corn stover has enhanced enzymatic hydrolysis and efficient removal of lignin. The analysis of variance of TRS mg/g after enzymatic saccharification is given in Table 7. In the Pareto chart, Figure 3A, the reference line 2.36 crosses the factors A, B, AA, and BB, representing the statistically significant factors. In Figure 3B, the dark green contour plot represents the region of maximum TRS production in the interaction between biomass loading and enzyme U (Sahare et al., 2012; Yang et al., 2022).

**TABLE 7:** Analysis of variance (ANOVA) of the results for the response of total reducing sugar (mg/g) after enzymatic saccharification.

Source	SS	Df	Mean square	F-value	*p*-value	
Model	5	385,511	77,102	324.15	0.000	Significant
Linear	2	359,244	179,622	755.16	0.000	
Biomass loading%	1	353,572	353,572	1,486.48	0.000	
Enzyme U	1	5,672	5,672	23.85	0.002	
Square	2	26,343	13,171	55.37	0.000	
Biomass loading% × Biomass loading	1	4,019	4,019	16.90	0.005	
Enzyme U × Enzyme U	1	19,543	19,543	82.16	0.000	
Two-way interaction	1	7	7	0.03	0.869	
Biomass loading × Enzyme U	1	7	7	0.03	0.869	
Error	7	1,665	238			
Lack of fit	3	1,173	391	3.18	0.146	
Pure error	4	492	123			
**Model statistics**						
R2	99.57					
Adj-R2	99.26					
Pred-R2	97.64					

### 3.5 PHA production by *Pseudomonas putida* using corn stover sugar hydrolysate

In this study, commercial cellulase was applied for enzymatic saccharification after alkali pretreatment of corn stover. The optimized condition obtained by RSM for the TRS was at 1.37% biomass loading and 44.85 U, which yielded 707.19 mg/g sugar. First, the growth of bacteria was tested in four conditions, as seen in Table 8, for growth curve, residual sugar (g/L), and DCW (g/L). The result indicates the maximum DCW (g/L) was obtained in D media conditions, which is modified media with hydrolysate (sodium chloride, ammonium sulfate, and hydrolysate). The dry cell weight is highest in modified medium with hydrolysate as corn stover hydrolysate is lignocellulosic, which is composed of D-glucose, D-xylose, L-arabinose, and other sugars (Baptista et al., 2018). As *Pseudomonas* is known for its metabolic versatility and adaptability, it utilizes different carbon sources and is efficient in capturing and metabolizing the hydrolysate components. In the case of modified media with glucose, it only uses one type of sugar and has less dry cell weight. Moreover, it is known that limiting nitrogen availability and providing an abundant carbon source induces PHA production, which clearly indicates the bacteria use excess carbon for PHA synthesis. All the above analysis demonstrates that nitrogen at 1 g/L and carbon (sugar) at 10 g/L, as given in our study, are favorable for PHA production.

**TABLE 8:** Cell growth of *Pseudomonas putida* in different media conditions.

S.no.	Media condition	Time h)	O.D.@600nm	Residual sugar (g/L)	DCW (g/L)
1	A	34	1.81 ± 0.006	1.19 ± 0.154	1.01 ± 0.000
2	B	34	0.86 ± 0.006	3.27 ± 0.007	0.45 ± 0.003
3	C	34	0.77 ± 0.003	5.19 ± 0.001	0.40 ± 0.07
4	D	34	2.47 ± 0.023	1.70 ± 0.015	1.41 ± 0.013

Modified media with hydrolysate was used as production media with ammonium sulfate supplemented to maintain nitrogen as a limiting factor with other components. In the production media (g/L), 1% sugar was used for PHA production from the sugar obtained after enzymatic saccharification. Only 1% sugar was used in the experiment as low sugar concentration decreases the growth of bacterial cells, which results in slow cell division. This favors the bacterial cells to use the resources for PHA synthesis rather than biomass accumulation. The carbon limitation stimulates a metabolic shift and enhances pathways to generate PHA as intracellular carbon and energy storage substances. Limiting sugar concentration optimizes the PHA production process and avoids the inhibitory role of high sugar concentrations. A high sugar concentration can be used for economic reasons for PHA production, but bench-scale research mainly focuses on low sugar concentrations to understand metabolic pathways and optimized conditions for PHA production (de Souza et al., 2020).

**FIGURE 4:**
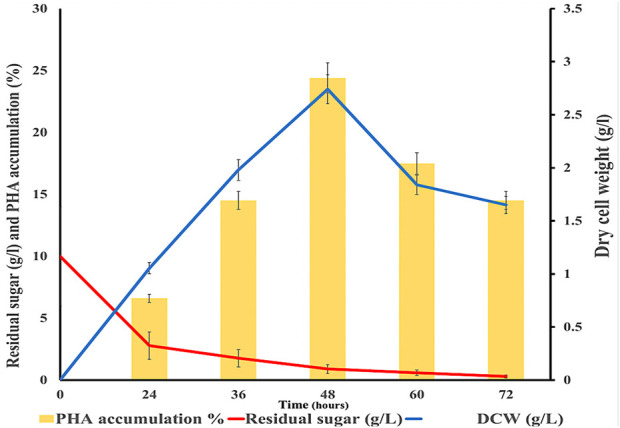
Graph showing dry cell weight (g/L), residual sugar (g/L), and PHA accumulation (%) in modified media with hydrolysate at different time intervals.

The maximum dry cell weight (DCW) was 2.74 g/L as shown in Figure 4. PHA accumulation of 24.4% and residual sugar of 0.89 g/L were observed at 48 h cultivation period, the corn stover hydrolysate used as a carbon source remained at 0.89%. Then, bacterial cells were harvested and further subjected to methanolysis for extraction of PHA. The PHA accumulation decreased after 48 h. There are many research reports on the major role of carbon and nitrogen in PHA production (Wang and Lee, 1997; Valentino et al., 2015). The limitation of nitrogen and the presence of carbon sources increase PHA productivity (Mahato, 2021).

Ashby et al. (2022) reported PHA production using corn stover hydrolysate and levulinic acid (Lev A). Corn stover was acid pretreated to recover sugars from the cellulosic and hemi-cellulosic fractions when processed with *Burkholderia sacchari* and the non-detoxified hydrolysate to achieve a maximum PHA titer of 1.2 g/L. Bacterium *Azohydromonas lata* induced bacterial growth and PHA production only in detoxified hydrolysate. Rebocho et al. (2019) used apple pulp waste from the fruit processing industry as feedstock for mcl-PHA production. *Pseudomonas citronellolis* has a polymer content of 30% wt. and PHA productivity of 0.025 g/l/h. The polymer is composed of 3-hydroxydecanoate (68% mol), 3-hydroxyactanoate (22% mol), 3-hydroxydodecanoate (5% mol), 3-hydroxytetradecanoate (4% mol), and 3-hydroxyhexanoate (1% mol), and its molecular weight is 3.7 × 10^5^ Da. It thermally degrades at 296°C. Thus, apple pulp is a suitable feedstock for mcl-PHA production. In a study by Lemechko et al. (2019), agro-industrial effluents were used for poly (3-hydroxybutyrate-co-hydroxyvalerate) by *Halomonas* sp. SF2003 with a PHA yield of 31% and PHA productivity of 1.89 g/L.

We can infer that lignocellulosic biomass (corn stover hydrolysate) is more favorable for the growth of microbial strain *P. putida* MTCC 2475 and the synthesis of biopolymer. The major challenge in the production of PHA is the high production cost, which can be highly reduced using easily available and renewable carbon sources. The results also demonstrate that enzymatic saccharification is favorable for enhanced sugar recovery. Table 9 denotes the different PHA contents found in other research studies and earlier reports using corn stover as hydrolysate. Therefore, corn stover is an economical and sustainable substrate for PHA content.

$$Residual\;biomass\;\left(g/L\right)=Dry\;biomass\;cell\;weight\;\left(g/L\right)-Extracted\;qiantity\;of\;PHA\;\left(g/l\right),$$

$$PHA\;accumulation\;\left(\%\right)=Extracted\;quantity\;of\;PHA\;\left(\frac{g}{L}\right)/\;Dry\;biomass\;cell\;weight\;\left(\frac{g}{L}\right)*100.$$

• ***A.*** *LB broth,* ***B.*** *only hydrolysate,* ***C.*** *Modified media with synthetic glucose,* ***D.*** *Modified media with hydrolysate.*
• **A.** Composition of LB broth (tryptone, sodium chloride, yeast extract), **B.** Only hydrolysate, **C.** Modified media with synthetic glucose (sodium chloride, ammonium sulfate, and glucose) and **D.** Modified media with hydrolysate (sodium chloride, ammonium sulfate, and hydrolysate).

**TABLE 9:** Summarized table of the different PHA contents in other research studies and earlier reports using corn stover as the hydrolysate.

S.no.	Substrate	Pretreatment/saccharification	Microorganisms for PHA production	Biopolymer	Polymer content	Reference
1	Corn stover hydrolysate	Alkaline pretreatment, densification, and enzyme digestibility	Metabolically engineered *Escherichia coli* WJ03-02	Poly (3-hydroxybutyrate-co-lactate)	19.00 wt%	Wu et al. (2021a)
2	Corn stover hydrolysate	1% (v/v) sulfuric acid pretreatment and absence of Lev A	*Azohydromonas lata* DSM 1122	Short-chain polyhydroxyalkanoate	No polymer production	Ashby et al. (2022)
3	Corn stover hydrolysate	1% (v/v) sulfuric acid pretreatment and absence of Lev A	*Burkholderia sacchari* DSM 17165	Short-chain polyhydroxyalkanoate	17.90%	Ashby et al. (2022)
4	Corn stover hydrolysate	Alkaline (3%sodium hydroxide) and enzymatic saccharification with cellulase from *Aspergillus niger*	*Pseudomonas putida* MTCC 2475	Medium-chain polyhydroxyalkanoate	24.40%	This study

### 3.6 Characterization of PHA

#### 3.6.1 GC-MS/MS and nuclear magnetic resonance

**FIGURE 5:**
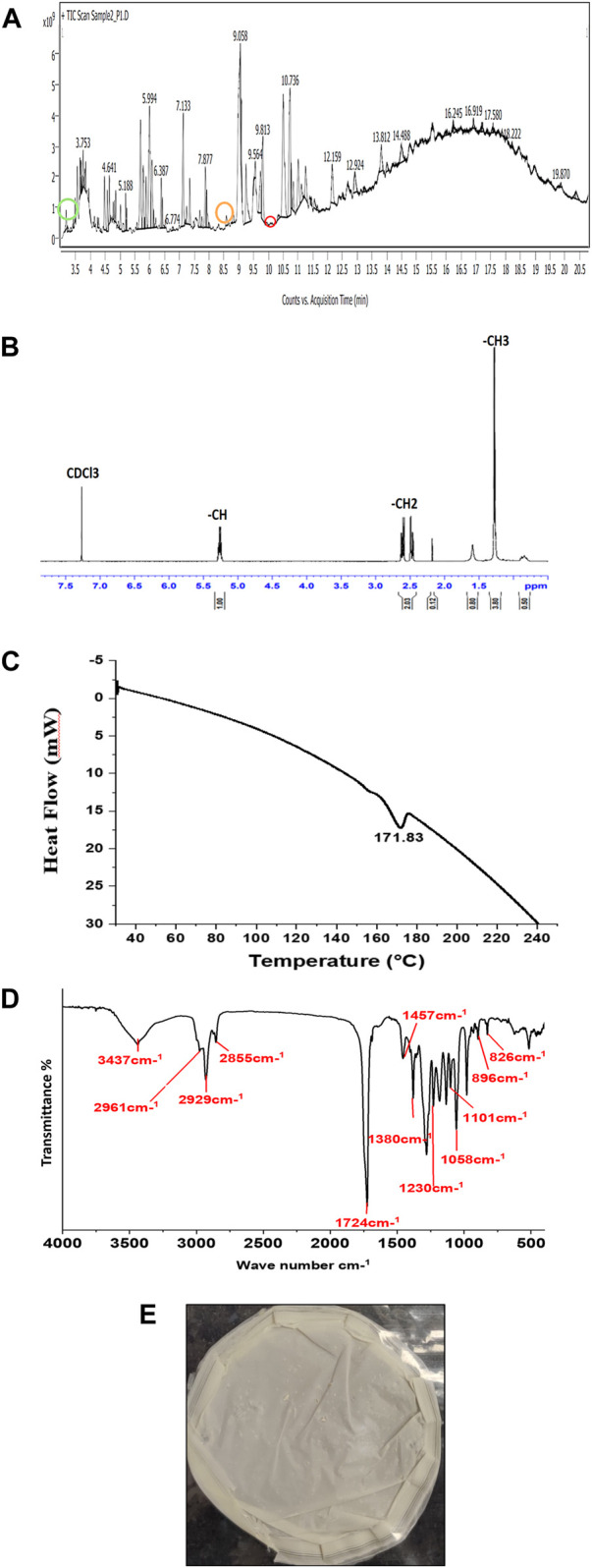
Characterization of PHA **(A)** GC-MS/MS chromatograms with three monomers of PHA (3-hydroxy octanoic acid methyl ester, 3-hydroxy tetra decanoate methyl ester, and 3-hydroxy dodecanoic acid methyl ester), **(B)** ^1^H NMR spectrum of PHA film from *Pseudomonas putida,* **(C)** DSC thermograms of the produced PHA film, **(D)** FTIR spectrum of extracted PHA film, and **(E)** solvent extracted PHA film from *Pseudomonas putida*.

The monomeric composition of PHA can be detected by GC-MS/MS and NMR. In the case of chromatography methods, the intact polymer cannot be detected, and depolymerization of the polymer, along with chemical derivatization, is necessary (Tan et al., 2014). PHA extracted from *P. putida* strain contained (3HO) 3-hydroxy octanoic acid methyl ester (6.40%), (3HTD) 3-hydroxytetradecanoate methyl ester (4.14%), and (3HDD) 3-hydroxydodecanoate methyl ester (2.79%). These compounds prove that monomers were present in the biodegradable polyester family and were confirmed through GC-MS/MS results, as shown in Figure 5A. The prominent peaks of 3- hydroxyoctanoic acid methyl ester (3HO), 3- hydroxydodecanoate acid methyl ester (3HDD) and 3-hydroxytetradecanoate acid methyl ester (3HTD) appeared at 3.183 min, 8.581 min, and 10.343 min, and their molecular formulas are C_9_H_18_O_3_, C_13_H_26_O_3_, and C_15_H_30_O_3_, respectively. *Pseudomonas* sp. mainly synthesizes mcl PHAs, which is proved by the three monomers present in the GC-MS/MS results. By gravimetric analysis, PHA accumulation was shown to be 24.40%. The NMR technique explains the intact PHA chemical structure and provides topology as well as functional groups in the molecule. ^1^H-NMR is sensitive, has high proton abundance in nature, and is performed in a short analytical time. NMR depicts saturated and unsaturated PHA analysis. According to reports in other research papers, peaks at 5.3 ppm correspond to the protons beside the carboxyl group, and chemical changes in the peak at 5.3 ppm specify the presence of an unsaturated group in the monomers (such as a methine group, -CH-). The spectrum shows a doublet at 2.5 ppm, comparable to the methylene group (-CH_2_). The peak at 1.6 ppm indicates the first CH_2_ of the side chain, and the peak at 1.3 ppm is the side chain CH_2_ group, such as the methyl group, as shown in Figure 5B. (Muangwong et al., 2016). According to a research study, GC-MS/MS results, along with the NMR spectrum, are pre-eminent analytical tools for investigating PHA (Thu et al., 2023).

#### 3.6.2 Differential scanning calorimetry

The thermal property of the extracted PHA film was analyzed by DSC, as shown in Figure 5C. Medium-chain length (mcl) polymers, as detected by GC-MS/MS in this research paper, are amorphous PHAs. They do not possess the ability to crystallize due to the chemical structure of PHAs. The benefits of amorphous PHAs are their flexibility and other mechanical strictures, similar to elastomers (Vostrejs et al., 2020). The extracted PHA film from *P. putida* has a melting temperature (Tm) peak of 171.8°C. The Tm values for PHA are in the range of non-observable to 177°C (Tan et al., 2014). The variation in the Tm depends on the composition of monomers in the biopolymer of PHA. The decomposition of the extracted PHA started at 200°C and lost its mass at 240°C (Sedlacek et al., 2020). The thermal degradation temperature (T_d_) for PHA is between 227°C and 256°C (Tan et al., 2014). The T_d_ of extracted PHA film is 240°C in our study.

#### 3.6.3 Fourier-transform infrared spectroscopy

The structural property of the extracted PHA film (Figure 5E) from *P. putida* was analyzed by FTIR, and the spectra were scanned from 4,000 to 400cm^−1^, as shown in Figure 5D. PHA biopolymers span from non-crystalline to highly crystalline. In the FTIR spectrum, PHA depicts characteristic infrared absorption bands at wavenumbers that indicate crystallinity (Tan et al., 2014). The presence of a broad characteristic peak of the hydroxyl group is at 3437 cm^−1^ (López-Cuellar et al., 2011). The presence of IR spectra at 2961 cm^−1^ and 2,926 cm^−1^ is due to the stretching of C-H of methyl and ethylene groups. The peak at 2855 cm^−1^ indicated –CH_2_-CH_3_. The prominent absorption bands at 1724 cm^−1^ and 1230 cm^−1^ show –C=O and –C-O-C- stretching (Tanikkul et al., 2020). Furthermore, the absorption band at 1724 cm^−1^ indicates the PHA marker band with the carbonyl C=O stretching vibrations of the ester group. The peaks at 1110 cm^−1^ and 797 cm^−1^ are due to vibrations in–C-O- and–C-C-, respectively (Sathiyanarayanan et al., 2017). Furthermore, according to Tanikkul et al., 2020, the peaks near 2,961 cm^−1^, 2,926 cm^−1^, 1724 cm^−1^, and 1058 cm^−1^ confirm the structure of mcl-PHA (Tanikkul et al., 2020). The presence of the–CH_3_ group at peak 1380 cm^−1^ is confirmed from the literature (Shamala et al., 2009).

**FIGURE 6:**
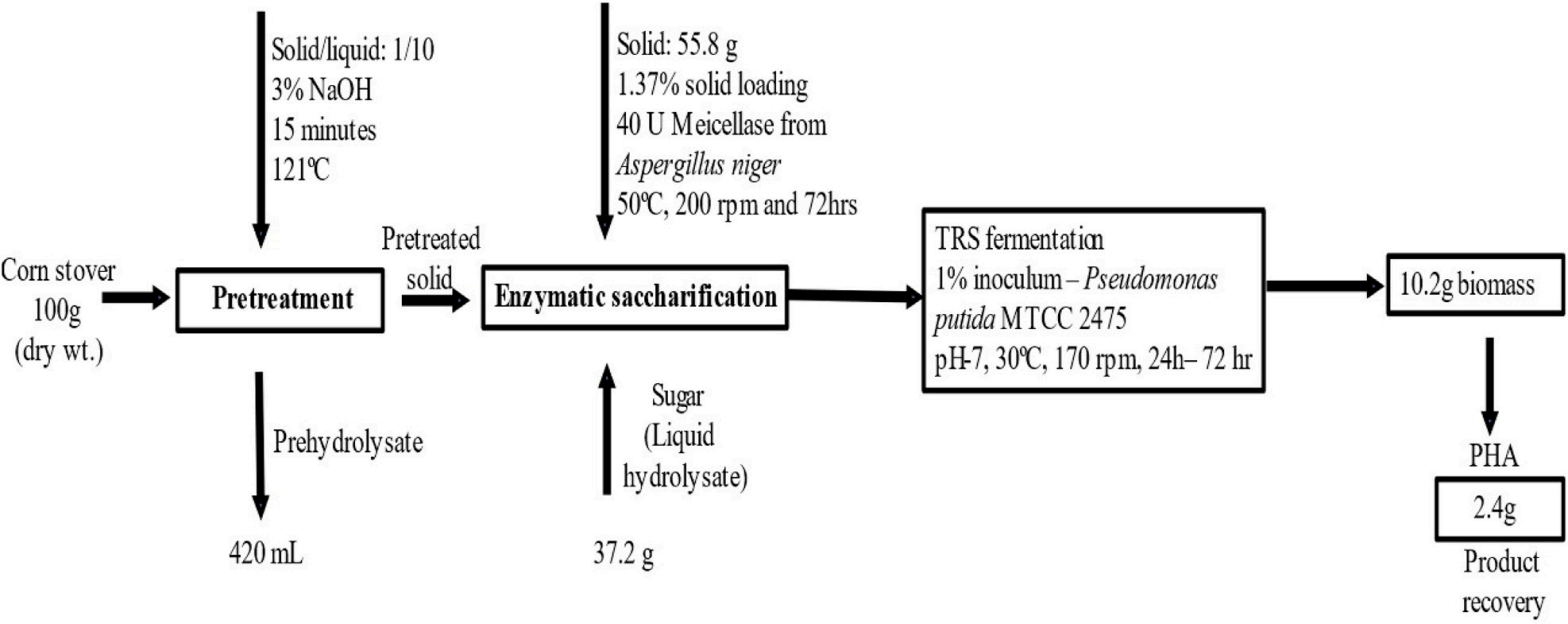
Overall steps of pretreatment, enzymatic saccharification, and fermentation and the mass balance study of using corn stover waste as a substrate for PHA production.

A mass balance study of utilizing corn stover as a substrate for PHA production was done to obtain and calculate the PHA derived from 100 g of corn stover, as shown in Figure 6. A 100 g sample of corn stover was pretreated with alkali, and 55.8 g of solid biomass was used for enzymatic saccharification using Meicellase from *A. niger* at 50°C, 200 rpm, and 72 h. The liquid hydrolysate was taken for fermentation using *P. putida* at 30°C, 170 rpm, and 48 h. Dry cell biomass recovered after fermentation was 10.2 g, and 2.4 g PHA was extracted. The major challenge in commercial PHA production is obtaining a high production, which is not met in this study due to the use of an inexpensive carbon source. However, the overall process of PHA production from corn stover is feasible as the raw material is globally produced agricultural waste. Finding an alternative, cheaper carbon source downsizes the cost of PHA production. Moreover, the wild-type *Pseudomonas putida* has stable genomes, less genetic instability, and optimizes production efficiency and can utilize different carbon sources such as readily available substrates.

## 4 Conclusion

PHA, an alternative to conventional plastic, is not widely produced due to its high cost. However, by optimizing the conditions of alkali pretreatment and enzymatic saccharification, corn stover stubble waste can be efficiently used to enhance the production of TRS. This sugar can be utilized by *P. putida* MTCC 2475 to synthesize PHA with a mix of different monomers. Therefore, corn stover waste is a renewable and readily available source for converting lignocellulosic biomass into reducing sugar, reducing the need for expensive substrates. Nevertheless, there is still a need for further research and optimization of production conditions at an industrial scale for PHA production.

## Data Availability

The original contributions presented in the study are included in the article/Supplementary Material; further inquiries can be directed to the corresponding author.
